# Pathway Towards High-Efficiency Eu-doped GaN Light-Emitting Diodes

**DOI:** 10.1038/s41598-017-15302-y

**Published:** 2017-11-07

**Authors:** Ioannis E. Fragkos, Chee-Keong Tan, Volkmar Dierolf, Yasufumi Fujiwara, Nelson Tansu

**Affiliations:** 1 Center for Photonics and Nanoelectronics, Department of Electrical and Computer Engineering, Lehigh University, Bethlehem, PA 18015 USA; 2 Department of Physics, Lehigh University, Bethlehem, PA 18015 USA; 3 Division of Materials and Manufacturing Science, Graduate School of Engineering, Osaka University, Suita, Osaka, 565-0871 Japan; 4 Department of Electrical and Computer Engineering, Clarkson University, Potsdam, NY 13699 USA

## Abstract

A physically intuitive current injection efficiency model for a GaN:Eu quantum well (QW) has been developed to clarify the necessary means to achieve device quantum efficiency higher than the state-of-the-art GaN:Eu system for red light emission. The identification and analysis of limiting factors for high internal quantum efficiencies (IQE) are accomplished through the current injection efficiency model. In addition, the issue of the significantly lower IQE in the electrically-driven GaN:Eu devices in comparison to the optically-pumped GaN:Eu devices is clarified in the framework of this injection efficiency model. The improved understanding of the quantum efficiency issue through current injection efficiency model provides a pathway to address the limiting factors in electrically-driven devices. Based on our developed injection efficiency model, several experimental approaches have been suggested to address the limitations in achieving high IQE GaN:Eu QW based devices in red spectral regime.

## Introduction

In recent years, III-Nitride based alloys have been placed at the frontiers of semiconductor technologies. The use of III-Nitrides has found places in a wide span of technological applications including bio-applications, thermoelectrics, solar cells, power electronics, optoelectronics and photonics^1–10^. Among III-Nitrides, InGaN-based alloys are of great interest. The versatility to tune the band-gap of InGaN-based alloys within the UV and visible spectral regime have established them as the main technologies driving the light emitting diode (LED) innovations^11–13^. The possibility for monolithically integrated red-green-blue (RGB) In_x_Ga_1−x_N-based LEDs, will also open a new era for smart and ultra-efficient solid stated lighting technology in the future^14^.

Despite the rapid development of high efficiency InGaN-based quantum well (QW) LEDs in the UV, blue and green spectral regime, the realization of InGaN QW-based LEDs with high efficiency at wavelengths towards to red spectra regime is challenging. The following issues associated with high indium content in the active region such as, phase separation of InGaN alloy and high polarizations fields, are detrimental to internal quantum efficiency (IQE) of the devices^15–19^. Several works in recent years have suggested innovative approaches with the potential of achieving high efficiency for InGaN based QW LED towards red spectral regime. These works include the investigation of staggered InGaN QWs, strain compensated InGaN QWs, alternative substrates and buffer layers of InGaN QWs, InGaN with AlGaN interlayer, semipolar and non-polar InGaN QW, InGaN-delta-InN QW and InGaN/dilute-As GaNAs interface QW^20–29^. Despite the demonstration of InGaN based LED in the red spectra regime, the highest reported external quantum efficiency (EQE) is 2.9% which is much lower from the blue and green InGaN-based QW LEDs^26^.

An alternative approach of achieving red emission without the need of high In-content of InGaN based alloys is the incorporation of rare earth elements into GaN (e.g Europium)^30–32^. The possibility of introducing Europium element (Eu) into the GaN material has enabled the realization of GaN:Eu red light emitting devices including LEDs in the past decade^33–49^. However, the internal quantum efficiency (IQE) of the GaN:Eu emitter is low (<1%), despite the recent years of effort in improving the device performance. These efforts include improving the GaN:Eu material quality and utilizing heterostructures for higher IQE^32,44,47–49^. Improving the IQE of the GaN:Eu devices is necessary for practical technological implementation. In addition, another major obstacle is found to be the IQE discrepancy between the electrically driven and optically excited GaN:Eu devices. Interestingly, the IQE of electrically-driven GaN:Eu devices is much lower than that of the optically-pumped GaN:Eu devices. Despite the fact that optically-pumped devices exhibited an increase in the output power and consequently in the IQE over the years, the electrically-driven devices showed a saturation in the output power, probably due to the IQE limitation of the device^50^. This discrepancy is possibly attributed to the dependency of the IQE on the current injection efficiency of the GaN:Eu active region for the two different excitation ways. The need of electrically-driven device is however arguably stronger than optically-pumped device because in many applications including LEDs, the devices are typically driven by injected current to achieve emission. If the GaN:Eu device is to be employed for the light emitting applications, the understanding of the factors which lead to low efficiency in electrically-driven GaN:Eu device needs to be enhanced. Thus, developing a current injection efficiency model of the GaN:Eu active region will provide a qualitative picture and a better understanding of the IQE of both optically-pumped and electrically-driven GaN:Eu red light emitters. Besides, the model can further provide the opportunity to enhance the design and fabrication of high efficiency GaN:Eu based red light emitters.

This work presents the development of physically intuitive current injection efficiency model for a GaN:Eu QW active region for understanding the discrepancy between the efficiencies of optically-pumped and electrically-driven GaN:Eu QW based devices. The discrepancies between the optically-pumped and electrically-driven RE-doped GaN LEDs devices can be explained from the differences on the carrier injection processes in the two types of devices. This study identifies and explains the limiting factors for the low IQE of the GaN:Eu QW active region and provides the pathway to enhance the IQE of the GaN:Eu QW based devices.

## Excitation path of Eu^+3^ ions in GaN:Eu QW and current injection efficiency models

### GaN:Eu QW active region considerations

The light emission from RE-doped semiconductors arises from the transitions between the intra 4 f electronic states of the RE ion. In the case of Eu-doped GaN semiconductor, the GaN acts as the host to the Eu^+3^ ions. The excitation of Eu^+3^ ion in GaN host is known to be mediated by traps which are present in the vicinity of Eu^+3^ ion. These traps capture free electron-hole pairs from GaN host and transfer the non-radiative recombination energy to a nearby Eu^+3^ ion^34,45,47,50–53^.

**Figure 1:**
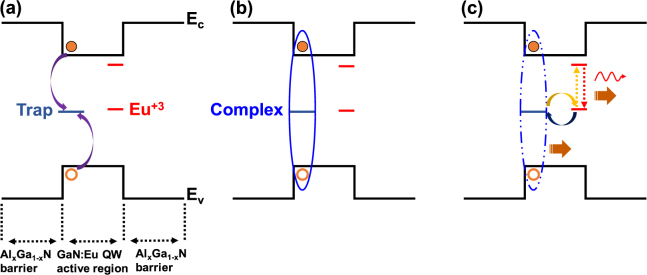
Model of the trap assisted excitation of Eu^+3^ ion in GaN:Eu QW active region. (**a**) The confined electron-hole in the GaN:Eu QW are captured by the traps (purple arrows) which are close to the vicinity of Eu^+3^ ion and results in (**b**) complex formation. (**c**) After the complex formation the electron-hole pair can recombine at the trap level by releasing a non-radiative energy to the crystal lattice (brown arrow) or release a non-radiative energy used for the excitation of the nearby Eu^+3^ ion (energy transfer process-gold arrow) or it can dissociate by releasing the electron-hole back to the GaN:Eu QW. Similarly, the excited Eu^+3^ can recombine non-radiatively by releasing energy to the crystal lattice (brown arrow) or release non-radiative energy for complex formation (energy back-transfer process, dark blue arrow) or recombine radiatively with photon emission (red arrow).

Our analysis in this work is carried out based on the model of a trap assisted excitation path of Eu^+3^ ion in the GaN:Eu QW active region with Al_x_Ga_1−x_N barriers as shown in Fig. 1. We represent the role of traps by a single trap level but note that the extended nature of these traps close to the vicinity of Eu^+3^ ions could also result in several levels. The electron-hole pair capture from the trap with a characteristic rate 1/τ_c_cap_ is notated as complex (bound-exciton) in our model, as shown in Fig. 1b. The subsequent recombination of carriers at the trap level (i.e. de-excitation of complex, Fig. 1c) releases a non-radiative recombination energy that leads to the following possible reactions: (a) excitation of the nearby Eu^+3^ ion with a characteristic energy transfer rate of 1/τ_tr,_ (b) energy to the crystal lattice with a characteristic rate of 1/τ_c_heat_. In addition, after the formation of complex, another process can occur which results in the electron-hole population of the QW with a characteristic rate of 1/τ_diss_ (complex dissociation process). Additional mechanisms to consider including the consequence of the then-de-excitation of Eu^+3^ ions: a) photon release with a characteristic rate of 1/τ_rad_, b) non-radiative de-excitation with a characteristic rate of 1/τ_Eu_heat_, and c) complex formation through energy back-transfer process with a characteristic rate of 1/τ_bt_. In addition, the carrier processes related to the GaN host and the Al_x_Ga_1−x_N barrier need to be taken into consideration, will be further discussed below.

### Electrical model

In the electrically-driven GaN:Eu QW device, carriers are injected into the GaN:Eu QW active region from the barriers. Our analysis is similar to the current injection efficiency analysis in a typical QW without the presence of RE elements^54,55^. The presence of Eu^+3^ ions modifies these rate equations to account for coupling with Eu^+3^ ions and complexes.

Previous experimental work on QW devices have shown that the carriers injected into the QW can escape to the barrier due to the high thermionic emission energy^56^. The thermionic-related carrier escape process needs to be accounted in the determination of IQE of electrically-driven QW based devices. In addition, the non-radiative and spontaneous radiative recombination process of carriers in the GaN host and Al_x_Ga_1−x_N barreirs are also taken into consideration in the electrically-driven GaN:Eu QW.

The carrier rate equations both in the barrier (N_B_) and GaN:Eu QW active region (N_QW_) are given by:

1 $$\frac{{{\rm{dN}}}_{{\rm{B}}}}{{\rm{dt}}}=\frac{{{\rm{I}}}_{{\rm{tot}}}}{{qV}_{{\rm{B}}}}+\frac{{{\rm{N}}}_{{\rm{QW}}}}{{{\rm{\tau }}}_{{\rm{e}}}}\frac{{V}_{{\rm{QW}}}}{{V}_{{\rm{B}}}}-{{\rm{N}}}_{{\rm{B}}}(\frac{1}{{{\rm{\tau }}}_{{\rm{B}}}}+\frac{1}{{{\rm{\tau }}}_{{\rm{bw}}}})$$

2 $$\frac{{{\rm{dN}}}_{{\rm{QW}}}}{{\rm{dt}}}=\frac{{{\rm{N}}}_{{\rm{B}}}}{{{\rm{\tau }}}_{{\rm{bw}}}}\frac{{V}_{{\rm{B}}}}{{V}_{{\rm{QW}}}}+\frac{{{\rm{N}}}_{{\rm{c}}}}{{{\rm{\tau }}}_{{\rm{diss}}}}\frac{{V}_{{\rm{Eu}}}}{{V}_{{\rm{QW}}}}-{{\rm{N}}}_{{\rm{QW}}}(\frac{1}{{{\rm{\tau }}}_{{\rm{nr}}}}+\frac{1}{{{\rm{\tau }}}_{{\rm{sp}}}}+\frac{1}{{{\rm{\tau }}}_{{\rm{e}}}}+\frac{1}{{{\rm{\tau }}}_{{\rm{c}}\_{\rm{cap}}}})$$

where, the V_B,_ V_QW_, V_Eu_ are the volumes of the barrier, GaN:Eu QW and Eu-doped region of the GaN:Eu QW respectively. The I_tot_ is the total injected current in the barriers which is assumed to be equal to the total injected current into the device, τ_e_ is the carrier thermionic escape time form the GaN:Eu QW active region to the barriers, τ_B_ is the carrier lifetime in the barrier described by the non-radiative and spontaneous radiative processes in the barrier, and τ_bw_ is the barrier-well lifetime^54,57^. The radiative and non-radiative carrier processes in the GaN host are described by the τ_sp_ and τ_nr_ respectively. In general, the non-radiative and spontaneous radiative recombination rates in the GaN host and Al_x_Ga_1−x_N barriers are functions of the carrier concentrations in the QW and barrier, the bimolecular recombination coefficient B, Shockley-Hall-Read (SHR) constant A, and Auger coefficient C. More details regarding the non-radiative and spontaneous radiative recombination processes of carriers in the GaN host and Al_x_Ga_1−x_N barriers, as well as the thermionic escape from GaN:Eu QW active region to the Al_x_Ga_1−x_N barriers can be found in refs^54,55,57–60^.

The rate equations of complexes (N_c_) and excited Eu^+3^ ions (N_Eu_) in the GaN:Eu QW active region are:

3 $$\begin{array}{rcl}\frac{{{\rm{dN}}}_{{\rm{c}}}}{{\rm{dt}}} & = & {{\rm{N}}}_{{\rm{QW}}}{{\rm{C}}}_{{\rm{c}}\_{\rm{cap}}}({{\rm{N}}}_{{\rm{traps}}}-{{\rm{N}}}_{{\rm{c}}})\frac{{V}_{{\rm{QW}}}}{{V}_{{\rm{Eu}}}}+{{\rm{N}}}_{{\rm{Eu}}}{{\rm{C}}}_{{\rm{bt}}}({{\rm{N}}}_{{\rm{traps}}}-{{\rm{N}}}_{{\rm{c}}})\\  &  & -{{\rm{N}}}_{{\rm{c}}}({{\rm{C}}}_{{\rm{tr}}}\,({\rm{N}}-{{\rm{N}}}_{{\rm{Eu}}})+\frac{1}{{{\rm{\tau }}}_{{\rm{diss}}}}+\frac{1}{{{\rm{\tau }}}_{{\rm{c}}\_{\rm{heat}}}})\end{array}$$

4 $$\frac{{{\rm{dN}}}_{{\rm{Eu}}}}{{\rm{dt}}}={{\rm{N}}}_{{\rm{c}}}{{\rm{C}}}_{{\rm{tr}}}({\rm{N}}-{{\rm{N}}}_{{\rm{Eu}}})-{{\rm{N}}}_{{\rm{Eu}}}({{\rm{C}}}_{{\rm{bt}}}({{\rm{N}}}_{{\rm{traps}}}-{{\rm{N}}}_{{\rm{c}}})+\frac{1}{{{\rm{\tau }}}_{{\rm{rad}}}}+\frac{1}{{{\rm{\tau }}}_{{\rm{Eu}}\_{\rm{heat}}}})$$

where, the N and N_traps_ are the concentrations of Eu^+3^ ions and traps in the GaN:Eu QW active region, respectively. The parameters C_c_cap_, C_bt_ and C_tr_ are defined as the capture, back-transfer and transfer coefficients in cm^3^/s respectively. For the rate equations (3) and (4), a general capture, back-transfer and transfer rate can be defined as:

5 $${{\rm{C}}}_{{\rm{c}}\_{\rm{cap}}}({{\rm{N}}}_{{\rm{traps}}}-{{\rm{N}}}_{{\rm{c}}})=\frac{1}{{{\rm{\tau }}}_{{\rm{cap}}0}}(1-\frac{{{\rm{N}}}_{{\rm{c}}}}{{{\rm{N}}}_{{\rm{traps}}}})=\frac{1}{{{\rm{\tau }}}_{{\rm{c}}\_{\rm{cap}}}}$$

6 $${{\rm{C}}}_{{\rm{bt}}}({{\rm{N}}}_{{\rm{traps}}}-{{\rm{N}}}_{{\rm{c}}})=\frac{1}{{{\rm{\tau }}}_{{\rm{bt}}0}}(1-\frac{{{\rm{N}}}_{{\rm{c}}}}{{{\rm{N}}}_{{\rm{traps}}}})=\frac{1}{{{\rm{\tau }}}_{{\rm{bt}}}}$$

7 $${{\rm{C}}}_{{\rm{tr}}}({\rm{N}}-{{\rm{N}}}_{{\rm{Eu}}})=\frac{1}{{{\rm{\tau }}}_{{\rm{tr}}0}}(1-\frac{{{\rm{N}}}_{{\rm{Eu}}}}{{\rm{N}}})=\frac{1}{{{\rm{\tau }}}_{{\rm{tr}}}}$$

Equations (5)–(7) account for saturation in the excited Eu^+3^ concentration as well as in the concentration of formed complexes, when substituted in the rate equations (3) and (4). The subscript 0 denotes the relative capture, transfer and back-transfer rate and the term in the parenthesis denotes the degree of the respective excitation of Eu^+3^ ion and the complex concentration. Thus, the terms of 1/τ_c_cap_, 1/τ_tr_ and 1/τ_bt_ can be viewed respectively as the general capture transfer and back-transfer rates of the system.

The injection efficiency of GaN:Eu QW active region is the ratio of the current arising from the radiative and non-radiative de-excitation of Eu^+3^ ions to the total current injected into the GaN:Eu QW system I_tot_, and can be expressed as:

8 $${{\rm{\eta }}}_{{\rm{inj}}\_{\rm{electrical}}}=\frac{{{\rm{I}}}_{{\rm{Eu}}}}{{{\rm{I}}}_{{\rm{tot}}}},$$

where, the I_Eu_ represents the total recombination current arising from the radiative and non-radiative de-excitation of the Eu^+3^ ion and is defined as:

9 $${{\rm{I}}}_{{\rm{Eu}}}=\frac{{{\rm{N}}}_{{\rm{Eu}}}{qV}_{{\rm{Eu}}}}{{\rm{\tau }}},$$

with

10 $$\frac{1}{{\rm{\tau }}}=\frac{1}{{{\rm{\tau }}}_{{\rm{rad}}}}+\frac{1}{{{\rm{\tau }}}_{{\rm{Eu}}\_{\rm{heat}}}},$$

where, the q is the electron charge.

Solving the system of equations (1)–(4) under steady state condition the current injection efficiency of the electrical model is obtained:

11 $${\eta }_{{\rm{i}}{\rm{n}}{\rm{j}}{\rm{\_}}{\rm{e}}{\rm{l}}{\rm{e}}{\rm{c}}{\rm{t}}{\rm{r}}{\rm{i}}{\rm{c}}{\rm{a}}{\rm{l}}}=[(\,1+\frac{{\tau }_{{\rm{b}}{\rm{w}}}}{{(\frac{1}{{\tau }_{{\rm{B}}}}+\frac{1}{{\tau }_{{\rm{b}}{\rm{w}}}})}^{-1}})(-\frac{\tau \,{\tau }_{{\rm{t}}{\rm{r}}}}{{\tau }_{{\rm{E}}{\rm{u}}}{\tau }_{{\rm{d}}{\rm{i}}{\rm{s}}{\rm{s}}}}+\frac{\tau \,{\tau }_{{\rm{c}}{\rm{\_}}{\rm{c}}{\rm{a}}{\rm{p}}}}{{(\frac{1}{{\tau }_{{\rm{n}}{\rm{r}}}}+\frac{1}{{\tau }_{{\rm{s}}{\rm{p}}}}+\frac{1}{{\tau }_{{\rm{c}}{\rm{\_}}{\rm{c}}{\rm{a}}{\rm{p}}}})}^{-1}}(\frac{{\tau }_{{\rm{t}}{\rm{r}}}}{{\tau }_{{\rm{E}}{\rm{u}}}\,{\tau }_{{\rm{c}}{\rm{o}}{\rm{m}}{\rm{p}}}}-\frac{1}{{\tau }_{{\rm{b}}{\rm{t}}}}))-\frac{\tau \,{\tau }_{{\rm{c}}{\rm{\_}}{\rm{c}}{\rm{a}}{\rm{p}}}}{{\tau }_{{\rm{e}}}}(\frac{{\tau }_{{\rm{t}}{\rm{r}}}}{{\tau }_{{\rm{E}}{\rm{u}}}\,{\tau }_{{\rm{c}}{\rm{o}}{\rm{m}}{\rm{p}}}}-\frac{1}{{\tau }_{{\rm{b}}{\rm{t}}}}){]}^{-1},$$

where, the 1/τ_Eu_ and 1/τ_comp_ are rates related to Eu^+3^ and complex:

12 $$\frac{1}{{{\rm{\tau }}}_{{\rm{Eu}}}}=\frac{1}{{{\rm{\tau }}}_{{\rm{rad}}}}+\frac{1}{{{\rm{\tau }}}_{{\rm{Eu}}\_{\rm{heat}}}}+\frac{1}{{{\rm{\tau }}}_{{\rm{bt}}}},$$

13 $$\frac{1}{{{\rm{\tau }}}_{{\rm{comp}}}}=\frac{1}{{{\rm{\tau }}}_{{\rm{tr}}}}+\frac{1}{{{\rm{\tau }}}_{{\rm{c}}\_{\rm{heat}}}}+\frac{1}{{{\rm{\tau }}}_{{\rm{diss}}}},$$

the internal quantum efficiency (η_IQE_electrical_) for the electrical model is given by:

14 $${{\rm{\eta }}}_{{\rm{IQE}}\_{\rm{electrical}}}={{\rm{\eta }}}_{{\rm{inj}}\_\mathrm{electrical}}\cdot {{\rm{\eta }}}_{{\rm{rad}}},$$

where, the η_rad_ is the radiative efficiency of the Eu^+3^ ions defined as the ratio of radiative to both radiative and non-radiative de-excitation of Eu^+3^ ions:

15 $${{\rm{\eta }}}_{{\rm{rad}}}=\frac{{{\rm{N}}}_{{\rm{Eu}}}{/{\rm{\tau }}}_{{\rm{rad}}}}{{{\rm{N}}}_{{\rm{Eu}}}/{\rm{\tau }}}=\frac{\frac{1}{{{\rm{\tau }}}_{{\rm{rad}}}}}{\,\frac{1}{{{\rm{\tau }}}_{{\rm{rad}}}}+\frac{1}{{{\rm{\tau }}}_{{\rm{Eu}}\_{\rm{heat}}}}}.$$

### Optical model

For the case of optically-pumped GaN:Eu QW, the thermionic emission rate from the GaN:Eu QW active region to the Al_x_Ga_1−x_N barrier is neglected. In optically-pumped GaN:Eu QW, the excitation of the GaN host is resonant, and the generated carriers do not possess excess energy to escape the QW^61–64^. For the same reason, the Al_x_Ga_1−x_N barriers are not excited and hence the non-radiative and radiative process of carriers in the barriers can be neglected.

In the optically-pumped GaN:Eu QW, the assumption that the GaN:Eu QW active region is excited resonantly above the bandgap with a photon flux φ, results in a rate equation of carriers in the GaN:Eu QW active region (N_QW_) of:

16 $$\frac{{{\rm{dN}}}_{{\rm{QW}}}}{{\rm{dt}}}={\rm{\alpha }}\,{\rm{\phi }}+\frac{{{\rm{N}}}_{{\rm{c}}}}{{{\rm{\tau }}}_{{\rm{diss}}}}\frac{{V}_{{\rm{Eu}}}}{{V}_{{\rm{QW}}}}-{{\rm{N}}}_{\mathrm{QW}}(\frac{1}{{{\rm{\tau }}}_{{\rm{nr}}}}+\frac{1}{{{\rm{\tau }}}_{{\rm{sp}}}}+\frac{1}{{{\rm{\tau }}}_{{\rm{c}}\_{\rm{cap}}}}),$$

where, the α is the absorption coefficient of GaN and the φ is the photon flux of the excitation. The first term of the right part of equation(16) can be viewed as the corresponding current I_tot_ arising from the creation of carriers due to absorption of the incident photon flux and is equal to:

17 $${\rm{a}}\,{\rm{\phi }}=\frac{{{\rm{I}}}_{{\rm{tot}}}}{{\rm{q}}\,{{\rm{V}}}_{{\rm{QW}}}}.$$

The rate equations of complexes (N_c_) and excited Eu^+3^ ions (N_Eu_) in the GaN:Eu QW active region are same as in the case of electrically-driven GaN:Eu QW and are given from equations (3)-(4). The injection efficiency for the optical model is defined as:

18 $${{\rm{\eta }}}_{{\rm{inj}}\_{\rm{optical}}}=\frac{{{\rm{I}}}_{{\rm{Eu}}}}{{{\rm{I}}}_{{\rm{tot}}}},$$

where, the I_Eu_ is defined from equation(9).

Solving the system of equations (3), (4) and (16) under steady state condition, the injection efficiency for the optical model is obtained:

19 $${{\rm{\eta }}}_{{\rm{inj}}\_{\rm{optical}}}=\,{(\frac{{\rm{\tau }}{{\rm{\tau }}}_{{\rm{c}}\_{\rm{cap}}}}{{(\frac{1}{{{\rm{\tau }}}_{{\rm{nr}}}}+\frac{1}{{{\rm{\tau }}}_{{\rm{sp}}}}+\frac{1}{{{\rm{\tau }}}_{{\rm{c}}\_{\rm{cap}}}})}^{-1}}(\frac{{{\rm{\tau }}}_{{\rm{tr}}}}{{{\rm{\tau }}}_{{\rm{Eu}}}{{\rm{\tau }}}_{{\rm{comp}}}}-\frac{1}{{{\rm{\tau }}}_{{\rm{bt}}}})-\frac{{\rm{\tau }}{{\rm{\tau }}}_{{\rm{tr}}}}{{{\rm{\tau }}}_{{\rm{Eu}}}{{\rm{\tau }}}_{{\rm{diss}}}})}^{-1}.$$

The internal quantum efficiency for the optical model is given from equation(14) with the respective injection efficiency.

### Comparison between optical and electrical model

**Figure 2:**
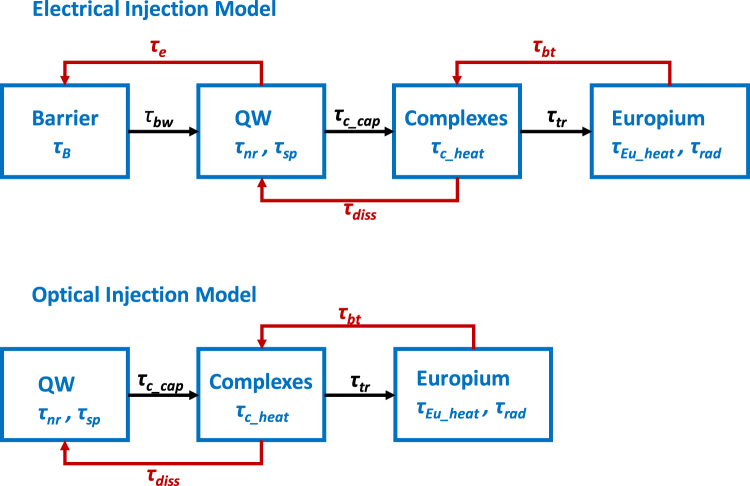
Flow charts of electrical and optical current injection efficiency models. The blue boxes indicate the different levels of barrier, GaN:Eu QW, complex and Eu^+3^ ion. Each level includes its own related processes. The levels are connected via the ‘forward mechanisms’ (black arrows), and via the ‘recycling mechanisms’ (red arrows).

The analysis of the current injection efficiency model indicates fundamental differences in the excitation path of Eu^+3^ ion in the GaN:Eu QW active region for the optically-pumped and electrically-driven GaN:Eu QW. In Fig. 2 a flow chart depicts the related mechanisms and phenomena along the excitation path of Eu^+3^ ion in the GaN:Eu QW for both models.

More specifically, the presence of the barrier level in the electrical model results in transport phenomena of the carriers. The effect of barrier-well lifetime which depends on the mobility of the carriers and the temperature T strongly influences the injection efficiency in the active region in a similar way as in the case of a QW without the presence of RE elements^55,60^. Additionally, recombination mechanisms (monomolecular, bimolecular and Auger recombination) also exist in the barrier. Further, the barrier opens an extra path for the carriers through the recycling mechanisms (red arrows in Fig. 2), increasing the probability of carrier deviation from the Eu^+3^ excitation path. The thermionic escape from QW to the barrier, which is proportional to the concentration of carriers (N_QW_), becomes stronger with increasing the current density^54,58,59^. The transport phenomena and thermionic process limit the injection efficiency and internal quantum efficiency in the electrically-driven GaN:Eu QW device as opposed to optically-pumped GaN:Eu QW in which these phenomena do not exist.

## Simulation Results

This section presents how the parameters such as SHR constant A, capture time τ_cap0_, transfer time τ_tr0_, back-transfer time τ_bt0_, dissociation time τ_diss_, and Eu^+3^ radiative lifetime τ_rad_, affect the injection efficiency of electrically driven and optical-pumped Eu-doped GaN QW active region. The QW and barrier parameters used for the simulations, such as the values of effective masses and mobilities, can be found in reference^57^. The bimolecular recombination coefficient B and Auger coefficient C are fixed to 10^−11^ cm^3^/s and 10^−32^ cm^6^/s respectively^57^. Note that the A, B and C coefficients, which describe the radiative and non-radiative processes in the GaN host and Al_x_Ga_1−x_N barriers, are assumed to be the same for the barriers and the well. The Al composition was set at x = 10% for the Al_x_Ga_1−x_N barriers. Table 1 presents the parameters used in the numerical calculation of the injection efficiency for the GaN:Eu QW active region. References^65,66^ were used as a starting point for the relative times between the GaN host, traps-complexes and Eu^+3^ ions.

**Table 1:** Parameters used for the numerical calculations of the current injection efficiency models.

Parameters	Study I	Study II	Study III	Study IV	Study V
A (10^7^ s^−1^)	0.1–1	1	1	1	1
τ_cap0_ (10^−7^ s)	10	0.1–10	10	10	10
τ_tr0_ (10^−7^ s)	360	360	3.6–360	360	360
τ_diss_ (10^−3^ s)	1	1	1	0.0001–1	1
τ_bt0_ (10^−6^ s)	200	200	200	2-200000	200
τ_c_heat_ (10^−3^ s)	1	1	1	1	1
τ_Eu_heat_ (10^−3^ s)	1	1	1	1	1
τ_rad_ (10^−6^ s)	400	400	400	400	30–400
N (cm^−3^)	1·10^19^	1·10^19^	1·10^19^	1·10^19^	1·10^19^
N_traps_ (cm^−3^)	1·10^19^	1·10^19^	1·10^19^	1·10^19^	1·10^19^
L_QW_, L_Eu_, L_B_ (nm)	2.5, 2.5, 5	2.5, 2.5, 5	2.5, 2.5, 5	2.5, 2.5, 5	2.5, 2.5, 5

Study of individual parameters associated with the Eu^+3^ excitation path.

**Figure 3:**
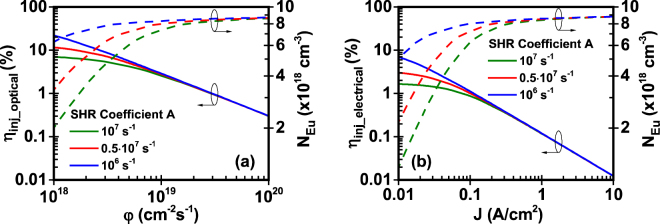
Effect of Shockley-Hall-Read constant A on injection efficiency and excited Eu^+3^ ion concentration of GaN:Eu QW active region. (**a**) Injection efficiency and excited Eu^+3^ ion concentration as a function of photon flux for optical model and (**b**) Injection efficiency and excited Eu^+3^ ion concentration as a function of current density for electrical model. The η_IQE_ is defined as η_IQE_ = η_inj_∙η_rad_ and follows the same trend as the η_inj_ of the optical and electrical model.

**Figure 4:**
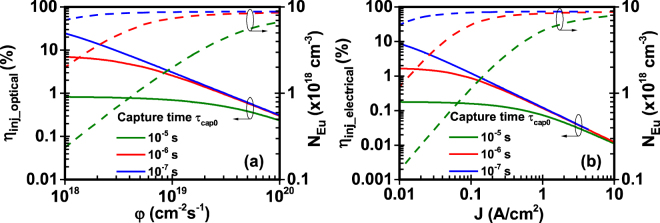
Effect of capture time τ_cap0_ on injection efficiency and excited Eu^+3^ ion concentration of GaN:Eu QW active region. (**a**) Injection efficiency and excited Eu^+3^ ion concentration as a function of photon flux for optical model and (**b**) Injection efficiency and excited Eu^+3^ ion concentration as a function of current density for electrical model. The η_IQE_ is defined as η_IQE_ = η_inj_∙η_rad_ and follows the same trend as the η_inj_ of the optical and electrical model.

**Figure 5:**
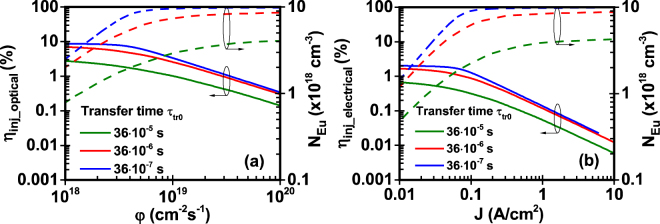
Effect of transfer time τ_tr0_ on injection efficiency and excited Eu^+3^ ion concentration of GaN:Eu QW active region. (**a**) Injection efficiency and excited Eu^+3^ ion concentration as a function of photon flux for optical model and (**b**) Injection efficiency and excited Eu^+3^ ion concentration as a function of current density for electrical model. The η_IQE_ is defined as η_IQE_ = η_inj_∙η_rad_ and follows the same trend as the η_inj_ of the optical and electrical model.

In our analysis, the injection efficiency (η_inj_optical_, η_inj_electrical_) is plotted with the excited Eu^+3^ concentration (N_Eu_) versus the photon flux (φ) - optical model -and input current density (J) - electrical model - (Figs 3–5). As shown in Figs 3–5, the injection efficiency of the Eu-doped GaN QW active region exhibits the droop characteristics. Since the excited Eu^+3^ concentration cannot exceed the maximum available Eu^+3^ concentration in the active region, the excited Eu^+3^ concentration increases with the photon flux and the current density. At a point where the excited Eu^+3^ concentration saturates due to the maximum available Eu^+3^ concentration in the active region, the subsequent increase of photon flux and current density leads to the droop in the injection efficiency. The rate of this saturation and the droop in the injection efficiency depend on the values of the different parameters related to specific mechanisms in the excitation path.

### Study I: Effect of Shockley-Hall-Read constant

The SHR constant A is related to the non-radiative process of monomolecular recombination which takes place through defects in the crystal lattice. SRH mechanism has been shown to be a critical process affecting the injection efficiency of light emitting diodes^57^. As shown in Fig. 3, at low photon fluxes and current densities, the injection efficiency is higher as the SRH constant is smaller. Such characteristic is expected, since lower values of SRH constant indicate lower non-radiative recombination rates of carriers in the active region and barrier. As a result, the injection efficiency in the GaN:Eu QW active region increases for optical and electrical model. Interestingly, it should be noted that the increase of the SHR constant A would lead to slower saturation of the excited Eu^+3^ concentration as the photon flux and current density is increasing. This indicates that additional carriers are required through optical excitation in the optically-pumped device or electrical injection in the electrically-driven device to replace the carriers lost in the monomolecular non-radiative recombination process. Thus, higher photon fluxes and current densities are required to result in same Eu^+3^ excitation as opposed to lower values of A.

### Study II: Effect of capture time

The capture of carriers from traps with a rate 1/τ_cap0_ results in the creation of complexes. A general capture time τ_c_cap_ is given from equation(5) which is a function of the formed complexes (N_c_). Figure 4 shows the effect of capture time τ_cap0_ both for optical and electrical model. Following the previous analysis, as the capture time decreases, the carriers are captured more efficiently from traps increasing the formation rate of complexes and consequently the excited Eu^+3^ concentration. This efficient capture of carriers from traps increases the injection efficiency and decrease the required amount of photon fluxes and current densities. This is observed as a shift towards lower photon fluxes and current densities of the excited Eu^+3^ concentration and injection efficiency for both models. For the optical model, the higher injection efficiency occurs for the lower capture time of τ_cap0_ = 10^−7^s where the injection efficiency drops from η_inj_optical_ = 21% to η_inj_optical_ = 0.2%. In contrast, for the electrical model it drops from η_inj_electrical_ = 9% to η_inj_electrical_ = 0.01%.

### Study III: Effect of transfer time

The transfer time defines the rate at which complexes de-excite by releasing energy to a nearby Eu^+3^ ion. As shown in Fig. 5, the injection efficiency increases as the transfer time τ_tr0_ decreases, which is a result of the faster de-excitation of the complexes. Equation(4) indicates that the de-excitation rate of complexes, 1/τ_tr_, is essentially the excitation rate of Eu^+3^ ions. As a result, the higher excitation rates of Eu^+3^ ions result in faster saturation of excited Eu^+3^ concentration under steady state conditions. This is observed as a shift toward lower photon fluxes (φ) and current densities (J) of the excited Eu^+3^ concentration. For the given range of photon flux and current density, the values of τ_tr0_ = 36 × 10^−6^ s and τ_tr0_ = 36 × 10^−7^ s result in saturation of excited Eu^+3^ concentration close to the value of Eu^+3^ ion concentration in the active region (N = 1 × 10^19^ cm^−3^), while the value of τ_tr0_ = 36 × 10^−5^ s results in saturation N_Eu_ = ~4 × 10^18^ cm^−3^ which is almost 40% of the total concentration of Eu^+3^ ion in the GaN:Eu QW active region.

### Study IV: Effect of complex dissociation rate and energy back-transfer rate

As stated before, the complexes can dissociate, releasing the captured electrons and holes into the QW with a rate of 1/τ_diss_. Similarly, the excited Eu^+3^ ions can de-excite with a rate 1/τ_bt0_ by releasing energy which results in the formation of complexes. Both dissociation time and back-transfer time are related to processes which can be considered as recycling mechanisms: in the case of dissociation process, the resulted electrons and holes can be re-captured from traps to form complexes, while in the back-transfer process the formed complexes can result to the excitation of Eu^+3^ ions. For this study, five different values of back-transfer and dissociation rates are selected for a given current density J = 0.87 A/cm^2^ and photon flux of φ = 4 × 10^19^ cm^−2^s^−1^.

**Figure 6:**
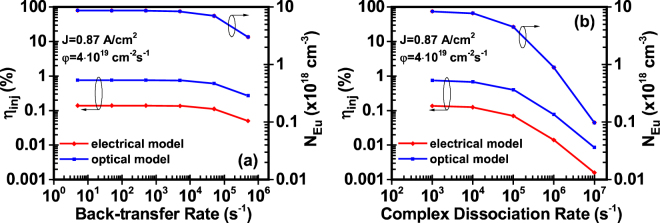
Injection efficiency and excited Eu^+3^ ion concentration of GaN:Eu QW active region as a function of (**a**) back-transfer rate 1/τ_bt0_ and (**b**) dissociation rate 1/τ_diss_. The η_IQE_ is defined as η_IQE_ = η_inj_∙η_rad_ and follows the same trend as the η_inj_ of the optical and electrical model. The two models are compared for the same values of Eu^+3^ excited ion concentration in the GaN:Eu QW active region.

As it is shown in Fig. 6, by increasing the dissociation rate, the injection efficiency and excited Eu^+3^ concnetration drop significantly. More specifically, for the electrical model injection efficiency drops from η_inj_electrical_ = 0.18% to almost η_inj_electrical_ = 0.001%, while for the optical model drops from η_inj_optical_ = 0.9% to almost η_inj_optical_ = 0.01%. The changes in excited Eu^+3^ concnetration are identical for the two models.

A droop in the injection efficiency and excited Eu^+3^ concentration with the back-transfer rate is also observed for both models. More specifically, the droop starts when the back-transfer rate of 1/τ_bt0_ = 5 × 10^4^ s^−1^ becomes comparable with the transfer rate of complexes, 1/τ_tr0_ = 2.77 × 10^4^ s^−1^. For back-transfer rates lower than 1/τ_bt0_ = 5 × 10^4^ s^−1^, the injection efficiency and excited Eu^+3^ concentration remain unaffected.

In addition, the changes in the injection efficiency and excited Eu^+3^ concnetration with the back-transfer rate, are smaller as compared to the changes with the complex dissociation rate. As it can be seen from Fig. 2, the level at which the dissociation process takes place is distant from the level of Eu^+3^ ion. Thus, the carriers resulted from the dissociation of complexes have higher probability to deviate from the Eu^+3^ excitation path reducing in that way the injection efficiency and the excited Eu^+3^ concentration in the GaN:Eu QW active region.

### Study V: Effect of radiative lifetime of Eu^+3^ ion – Enhancement of radiative efficiency

The parameters presented in the previous sections affect the internal quantum efficiency of the system by altering only the injection efficiency in the active region. The internal quantum efficiency is calculated from equation(14) with a radiative efficiency fixed at η_rad_ = ~72% and follows the same trend of the injection efficiency. The radiative lifetime (τ_rad_) and the non-radiative time (τ_Eu_heat_) of Eu^+3^ ion determine the radiative efficiency of the GaN:Eu QW system. Lower radiative lifetime results in higher radiative efficiencies, assuming that the non-radiative lifetime of Eu^+3^ ion remains unchanged.

**Figure 7:**
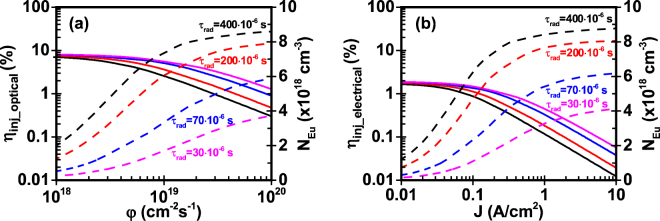
Effect of radiative lifetime τ_rad_ of Eu^+3^ ion on injection efficiency and excited Eu^+3^ ion concentration of GaN:Eu QW active region. (**a**) Injection efficiency and excited Eu^+3^ ion concentration as a function of photon flux for optical model and (**b**) Injection efficiency and excited Eu^+3^ ion concentration as a function of current density for electrical model. The η_IQE_ follows the same trend as the η_inj_ for the optical and electrical model. The non-radiative lifetime of Eu^+3^ ion is set to τ_Eu_heat_ = 1 ms. Different radiative lifetimes correspond to different radiative efficiencies. For τ_rad_ = 400 μs the radiative efficiency is η_rad_ = 71.43%. Similarly, for τ_rad_ = 200 μs / η_rad_ = 83.3%, for τ_rad_ = 70 μs / η_rad_ = 93.46%, and for τ_rad_ = 30 μs / η_rad_ = 97.09%.

By reducing the radiative lifetime, the injection efficiency and excited Eu^+3^ concentration are significantly altered. The lower radiative lifetime indicates faster radiative de-excitation rate of excited Eu^+3^ ions, therefore, higher injection efficiency can be achieved at a given photon flux and current density. This is clearly illustrated in Fig. 7. In addition, the resulted lower saturation values of excited Eu^+3^ ions, make the injection efficiency to be strongly altered at higher photon fluxes and current densities.

Attributing to the differences in complex interplays among the fundamental processes in the current injection process, the optical model exhibits higher injection efficiency as compared to the electrical model for the same values of excited Eu^+3^ concentration. In particular, the reduction of radiative lifetime from τ_rad_ = 400 μs to τ_rad_ = 30 μs, changes the excited Eu^+3^ concentration from N_Eu_ = 8.4 × 10^18^ cm^−3^ to N_Eu_ = 3.25 × 10^18^ cm^−3^ at a given φ = 4.7 × 10^18^ cm^−3^. Meanwhile, injection efficiency increases from η_inj_optical_ = 0.62% to η_inj_optical_ = 2.4% which is 3.8 times higher. A similar change in the excited Eu^+3^ concentration occurs at J = 1 A/cm^2^ for the electrical model while the injection efficiency increases from η_inj_electrical_ = 0.12% to η_inj_electrical_ = 0.46% which is almost 3.8 times higher, same change as in the optical model. The reduction of the radiative lifetime is essential for achieving higher injection efficiencies at higher photon fluxes and current densities, while at the same time the radiative efficiency of Eu^+3^ ions is enhanced.

## Comparison with experimentally reported data

In order to compare our work with experimentally reported values of GaN:Eu devices, the external quantum efficiency (η_EQE_) for a GaN:Eu QW LED with a square device area of 1000 × 1000 μm is calculated. The external quantum efficiency is the product of the extraction efficiency (η_extr_) and the internal quantum efficiency of the device. An extraction efficiency of η_extr_ = 44% was used for our calculations, which is a typical value for GaN:Eu based device^48^. The details of each simulation are given in Table 2a and b. The numerical calculations for the external quantum efficiency are divided into two groups: Group A represents those which resulted in η_EQE_ > 1% and Group B represents those which resulted in η_EQE_ < 1%.

**Table 2:** (a) Simulations of external quantum efficiency (EQE) for a GaN:Eu QW device-high EQE. (b) Simulations of external quantum efficiency for a GaN:Eu QW device-low EQE.

Parameters	Simulation I	Simulation II	Simulation III	Simulation IV
(**a**)				
A (s^−1^)	0.5·10^8^	10^6^	10^6^	10^6^
τ_cap0_ (s)	10^−7^	10^−7^	10^−8^	10^−8^
τ_tr0_ (s)	36·10^−6^	36·10^−6^	36·10^−7^	36·10^−7^
τ_diss_, τ_bt0_ (s)	10^−3^, 200·10^−6^	10^−3^, 200·10^−6^	10^−3^, 200·10^−6^	10^−3^, 200·10^−6^
τ_c_heat_, τ_Eu_heat_ (s)	10^−3^, 10^−3^	10^−3^, 10^−3^	10^−3^, 10^−3^	10^−3^, 10^−3^
τ_rad_ (s)	200·10^−6^	200·10^−6^	200·10^−6^	100·10^−6^
N (cm^−3^)	8.5·10^19^	8.5·10^19^	8.5·10^19^	8.5·10^19^
N_traps_ (cm^−3^)	8.5·10^19^	8.5·10^19^	8.5·10^19^	8.5·10^19^
L_QW_, L_Eu_, L_B_ (nm)	5, 5, 10	5, 5, 10	5, 5, 10	5, 5, 10
(**b**)				
A (s^−1^)	10^6^	10^6^	10^6^	10^6^
τ_cap0_ (s)	10^−4^	10^−6^	10^−6^	10^−6^
τ_tr0_ (s)	36·10^−6^	36·10^−6^	36·10^−4^	36·10^−6^
τ_diss_, τ_bt0_ (s)	10^−3^, 200·10^−6^	10^−6^, 200·10^−6^	10^−3^, 200·10^−6^	10^−3^, 200·10^−8^
τ_c_heat_, τ_Eu_heat_ (s)	10^−3^, 10^−3^	10^−3^, 10^−3^	10^−3^, 10^−3^	10^−3^, 10^−3^
τ_rad_ (s)	200·10^−6^	200·10^−6^	200·10^−6^	100·10^−6^
N (cm^−3^)	8.5·10^19^	8.5·10^19^	8.5·10^19^	8.5·10^19^
N_traps_ (cm^−3^)	8.5·10^19^	8.5·10^19^	8.5·10^19^	8.5·10^19^
L_QW_, L_Eu_, L_B_ (nm)	5, 5, 10	5, 5, 10	5, 5, 10	5, 5, 10

**Figure 8:**
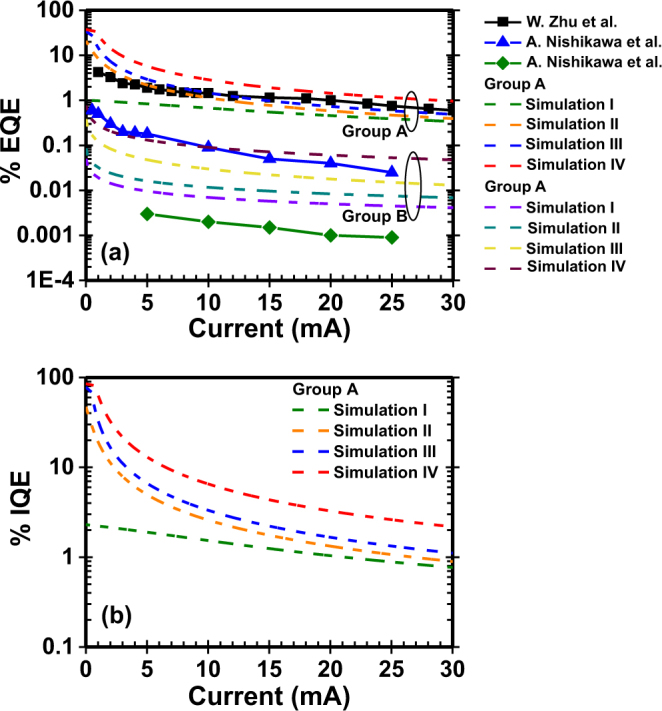
(**a**) EQE calculated from the electrical current injection efficiency model of GaN:Eu QW device and experimentally reported values of GaN:Eu based LED. (**b**) Calculated IQE for the electrically-driven GaN:Eu QW device. The simulation parameters are shown in Table 2(a).

Figure 8(a) presents our numerical calculations and experimentally reported values with two different types of GaN:Eu based LED. A. Nishikawa and co-workers fabricated two GaN:Eu based LEDs with a 300 nm GaN:Eu active layer each, under different growth conditions^44^. They reported an external quantum efficiency of η_EQE_ = 0.6% at an injected current of 0.5 mA which was found to reduce to η_EQE_ = 0.04% at 20 mA. W. Zhu and co-workers fabricated a GaN:Eu based LED with an active layer of alternate GaN/GaN:Eu regions and they reported an external quantum efficiency of η_EQE_ = 4.6% at an injected current of 1 mA which reduced to η_EQE_ = 0.9% at 20 mA^49^. These values correspond to the highest reported external quantum efficiency up to date. The calculated EQE from the electrical current injection efficiency model, follows the same trend as the experimentally reported values.

In addition, both experimental studies revealed that higher injected current into the GaN:Eu device led to saturation in the EL spectra, which was attributed to the saturation of the excited Eu^+3^ ions. Similar findings have also been reported elsewhere^45,47^. Our study is consistent with the experimental observations that increasing the injected current will eventually result in the saturation of the excited Eu^+3^ concentration with a subsequent decrease in the injection efficiency and internal quantum efficiency the GaN:Eu QW active region.

## Engineering the IQE of electrically-driven GaN:Eu QW

The increase of the injection efficiency, as well as its shift at higher input current densities is the desirable goal for highly efficient electrically-driven GaN:Eu based red light emitters. The numerical calculations from the current injection efficiency model, showed the pathway for high efficiency in the GaN:Eu QW system. By physically engineering the factors in the GaN:Eu QW system that affect injection efficiency (η_inj_) is thus critical for achieving high internal quantum efficiency (η_IQE_) in electrically-driven GaN:Eu QW based devices.

### Material quality

The SHR constant A is related to defects present in the barrier and QW. Higher values of A reflect poor material quality which is detrimental for IQE of the device. The numerical calculation of IQE of the electrically-driven GaN:Eu QW device, showed an increase 62% in the IQE at 5 mA when the SHR constant A decreased by 98% (Fig. 8(b), Simulation I and Simulation II). High quality Al_x_Ga_1−x_N alloy material with low defect concentration can be fabricated with advanced growth techniques, such as MOCVD by carefully adjusting the growth parameters^67–70^. This low defect concentration suppresses the SHR mechanisms and is expected to increase the IQE of GaN:Eu QW based device.

### Carrier capture process, back-transfer process and complex related processes

The capture time of carries form traps, the lifetime parameters related to complexes and the back-transfer process, depend on the nature of those elements as well as on the interaction between them and with the host. This work quantitatively verified that a significant enhancement of the IQE of the electrically-driven GaN:Eu QW device can be achieved by changing these lifetimes. A decrease of 90% both in capture time and transfer time resulted in an IQE increase of ~33% at 5 mA and ~24% at 20 mA (Fig. 8(b), Simulation II and Simulation III). Recent studies have shown that through defect engineering such as co-doping with magnesium (Mg), and also through manipulation of growth conditions, can result in the enhancement of the IQE of GaN:Eu device^44,49,50,52,53,71^.

### Europium and trap concentration in the GaN host and thermionic emission process

Another parameter that can be engineered is the number of available traps (N_traps_) and Eu^+3^ ion concentration (N). In this work, the effect of these two parameters is not presented. However, these values can be modified to increase the injection efficiency. More specifically, by increasing the amount of available traps (N_traps_), the general capture rate according to equation(5) will be increased giving rise to the injection efficiency. Similarly, the simultaneous increase of Eu^+3^ ion concentration (N) will also increase the transfer rate according to equation(7). As a result, the injection efficiency will be increased at higher input current densities and photon fluxes.

In our study the Al composition of the barrier was set to 10%. Higher Al composition will increase the conduction and valence band offsets and will suppress the carrier thermionic escape the barrier^57^. Thus, engineering the barrier height for carriers is crucial for higher injection efficiencies in the GaN:Eu QW active region.

### Radiative lifetime of Eu^+3^ ion

The radiative efficiency of Eu^+3^ ion can also be modified through the engineering of radiative lifetime of Eu^+3^ ions. Our numerical calculations of IQE of the electrically-driven GaN:Eu QW device, showed that changing the radiative lifetime from τ_rad_ = 200 μs to τ_rad_ = 100 μs results in an increase of ~144% of the IQE at 20 mA of the GaN:Eu QW device (Fig. 8(b), Simulation III and Simulation IV). It has been experimentally demonstrated that by utilizing surface-plasmon (SP) in GaN-based QW can dramatically increase the radiative efficiency of the system^72–75^. The photon density states near the SP frequency (ϖ_sp_) are increased from Purcell factor. For the case of GaN:Eu QW system, by carefully engineering the deposited materials used as SP, the SP frequency can be adjusted to coincides with the frequency of the emitted photons of Eu^+3^ ions. This approach will increase the radiative efficiency and consequently the internal quantum efficiency of the GaN:Eu based devices.

## Summary

In summary, we developed a physically intuitive current injection efficiency model for optically-pumped and electrically-driven GaN:Eu QW and we demonstrated the pathway for enhancing the internal quantum efficiency (η_IQE_) of a GaN:Eu QW system. It was shown that the saturation of excited Eu^+3^ concentration with the photon flux and input current density, is the main cause of the current injection efficiency droop in the GaN:Eu QW active region. Through the manipulation of the characteristic times along the excitation path of Eu^+3^ ion, the injection efficiency (η_inj_) and internal quantum efficiency (η_IQE_) of GaN:Eu QW system can be significantly enhanced. In addition, the discrepancy between the efficiencies of optically-pumped and electrically-driven GaN:Eu QW is explained in the framework of the current injection efficiency models. Our findings through the analysis within the current injection efficiency model also clarify the necessary means towards the practical realization of highly efficient red light GaN:Eu QW LED.
